# Exercise-mimetic AICAR transiently benefits brain function

**DOI:** 10.18632/oncotarget.4715

**Published:** 2015-07-17

**Authors:** Davide Guerrieri, Henriette van Praag

**Affiliations:** ^1^ Neuroplasticity and Behavior Unit, Laboratory of Neurosciences, National Institute on Aging, Baltimore, MD 21224

**Keywords:** Gerotarget, running, AMPK, AICAR, hippocampus, muscle

## Abstract

Exercise enhances learning and memory in animals and humans. The role of peripheral factors that may trigger the beneficial effects of running on brain function has been sparsely examined. In particular, it is unknown whether AMP-kinase (AMPK) activation in muscle can predict enhancement of brain plasticity. Here we compare the effects of running and administration of AMPK agonist 5-Aminoimidazole-4-carboxamide 1-β-D-ribofuranoside (AICAR, 500 mg/kg), for 3, 7 or 14 days in one-month-old male C57BL/6J mice, on muscle AMPK signaling. At the time-points where we observed equivalent running- and AICAR-induced muscle pAMPK levels (7 and 14 days), cell proliferation, synaptic plasticity and gene expression, as well as markers of oxidative stress and inflammation in the dentate gyrus (DG) of the hippocampus and lateral entorhinal cortex (LEC) were evaluated. At the 7-day time-point, both regimens increased new DG cell number and brain-derived neurotrophic factor (BDNF) protein levels. Furthermore, microarray analysis of DG and LEC tissue showed a remarkable overlap between running and AICAR in the regulation of neuronal, mitochondrial and metabolism related gene classes. Interestingly, while similar outcomes for both treatments were stable over time in muscle, in the brain an inversion occurred at fourteen days. The compound no longer increased DG cell proliferation or neurotrophin levels, and upregulated expression of apoptotic genes and inflammatory cytokine interleukin-1β. Thus, an exercise mimetic that produces changes in muscle consistent with those of exercise does not have the same sustainable positive effects on the brain, indicating that only running consistently benefits brain function.

## INTRODUCTION

Consistent evidence is emerging from both human and animal research that exercise benefits brain function throughout the lifespan. Physical activity may also translate into prevention or delay of neurodegenerative disorders [1, 2]. In the brain, underlying mechanisms that have been investigated include neurotransmitters, neurotrophins, fine neuronal morphology, blood flow, angiogenesis and hippocampal neurogenesis [1, 3]. In particular, the dentate gyrus (DG) of the hippocampus, a region vulnerable to neurodegeneration [4] and crucial for new memory formation, is positively influenced by exercise [3, 5]. Voluntary wheel running increases new DG neuron production [3], reduces hippocampal neuroinflammation [6, 7] and elevates neurotrophin levels, such as brain-derived neurotrophic factor (BDNF) [8]. BDNF signaling is involved in cell survival, synaptogenesis and cognitive function [1, 9]. Moreover, running up-regulates BDNF in the perirhinal cortex [10], a brain area that, together with the lateral entorhinal cortex (LEC), provides significant afferent input to adult-born DG neurons [11].

Interestingly, the peripheral triggers that may elicit the response of the central nervous system to running remain unclear. Blocking systemic neurotrophic factors such as insulin-like growth factor (IGF) and vascular endothelial growth factor (VEGF) preclude exercise-induced neurogenesis [12, 13]. Muscle activation may result in release of myokines [14] that influence other organs, including the brain. Muscle energy metabolism is regulated by AMP-kinase (AMPK), a key energy-sensing enzyme that is activated by a decrease in the ATP/AMP ratio within cells [15]. AMPK lies at the core of complex interconnected energy-sensing networks that include other transcriptional co-activators, such as peroxisome proliferator-activated receptor gamma coactivator-1 alpha (PGC-1α) [16]. Activation of this metabolic network [17] increases catabolism [18], and reduces anabolic processes [19]. Pharmacological or transgenic activation of these transcription factors in muscle can mimic effects of exercise on endurance [20], cognition, adult neurogenesis [21, 22], and mood [23]. Conversely, lack of functional AMPK in skeletal muscle precludes spatial memory improvement [22].

It remains unclear how AMPK activation in muscle affects brain function. In previous studies AMPK agonist 5-Aminoimidazole-4-carboxamide 1-β-D-ribofuranoside (AICAR) improved memory function and neurogenesis when administered for one week, but not upon longer treatment [21]. Therefore, we compared the time-course of peripheral and central effects of AICAR and exercise, measuring energy pathway activation in muscle and indices of hippocampal and cortical neural plasticity. In particular, we aimed to determine effects of AICAR and running on cell proliferation [21, 24], BDNF levels [8, 9], inflammatory cytokines [6, 7, 25], oxidative stress [26, 27], and gene expression in DG and LEC. Our results show that, in muscle, effects of AICAR and exercise overlap, while in the brain, cell proliferation, neurotrophin levels and expression of neural plasticity-relevant genes are only transiently increased by AICAR. Indeed, fourteen days of AICAR resulted in increased expression of apoptotic genes and inflammatory cytokine levels, and differentially regulated oxidative stress markers. Our data indicate that prolonged pharmacological activation of muscle energy metabolism may hamper brain function.

## RESULTS

### AICAR and running increase AMPK pathway proteins in muscle

**Figure 1:**
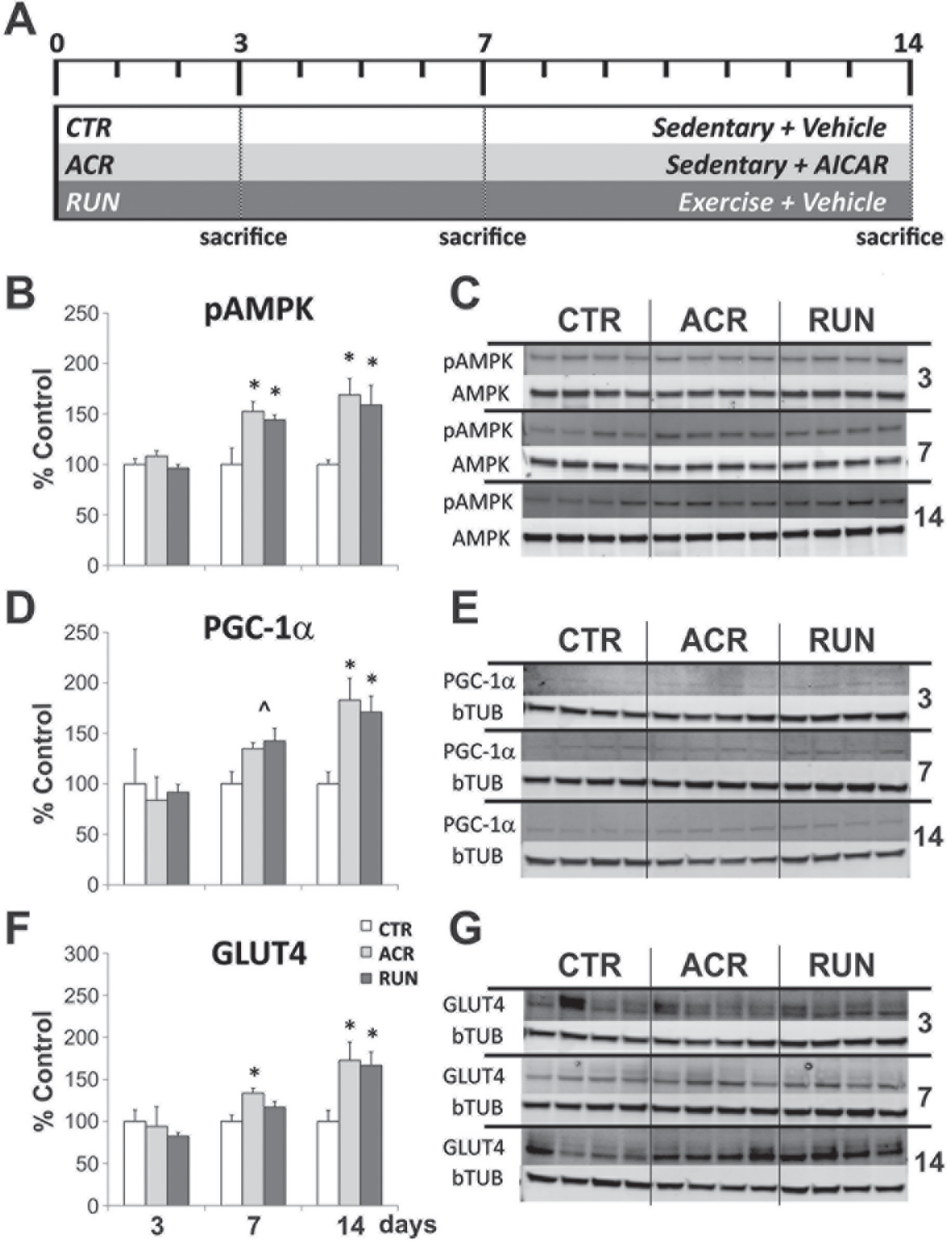
Comparison between effects of AICAR and running on expression levels of AMPK pathway components in muscle **A.** Timeline of the running and AICAR treatment. CTR = control groups; ACR = AICAR treated groups; RUN = voluntary running groups. Immunoblotting of gastrocnemius tissues after 3, 7 and 14 days of treatment; **B–C.** Phosphorylation of AMPK is increased by both AICAR and running after 7 days of treatment; **D–E.** Expression levels of PGC-1α showed a trend towards an increase after both treatments after 7 days, and a significant increase after 14 days; **F–G.** Expression levels of GLUT4 are increased by AICAR after 7 days, and by both AICAR and running after 14 days. (*p < 0.05; ^˄^*p* = 0.066). Error bars denote S.E.M.

To compare effects of AICAR and exercise in muscle, we evaluated the activation of the AMPK pathway. Specifically, the phosphorylation levels of AMPK (pAMPK), and the expression levels of PGC-1α and Glucose Transporter type 4 (GLUT4) were measured. One-month-old male mice were divided into control (CTR), AICAR (ACR) and exercise (RUN) groups (*n* = 7–8 per group) and evaluated at three different time points (3, 7 and 14 days). All animals received daily saline (CTR, RUN) or AICAR (ACR) injections (Figure 1A, Table 1). The RUN groups had free access to running wheels; daily running distances were not significantly different (F_(2, 26)_ = 0.73, *p* = 0.49) between RUN3 (3420 ± 726 m/day), RUN7 (3182 ± 381 m/day) and RUN14 (2649 ± 329 m/day) groups. Gastrocnemius muscle tissue was used for western blot analysis.

**Table 1:** Mouse age, groups and treatment duration

**MOUSE AGE**	4 weeks, 3 days			5 weeks			6 weeks		
**TREATMENT DURATION**	3 days			7 days			14 days		
**TREATMENT**	Control	AICAR	Exercise	Control	AICAR	Exercise	Control	AICAR	Exercise
**GROUP (N)**	4	4	4	4/8^1^	4/8^1^	4/8^1^	4/8^1^	4/7^1^	4/7^1^

Mice were divided into Control, AICAR or Exercise groups. Treatment lasted 3, 7 or 14 days, and mouse age at the completion of treatment is shown in the top row.

1 animals injected with bromodeoxyuridine (BrdU), 50 mg/kg, daily for seven days.

At the 3-day time-point no changes were observed in any of the proteins (pAMPK, PGC-1α, GLUT-4) measured (Figure 1B–1G). One way analysis of variance (ANOVA) and Fisher's post-hoc analysis showed a significant increase in muscle pAMPK levels after 7 days for AICAR treated and running mice (F_(2, 20)_ = 6.72, *p* < 0.006). At the 7-day time-point specific post-hoc comparisons showed that both ACR7 (155 ± 9%) and RUN7 (135 ± 3%) differed significantly from CTR7 (100 ± 16%; *p* < 0.05). After 14 days both treatments also showed an increase of pAMPK levels (F_(2, 19)_ = 7.35, *p* < 0.004) compared to the control group. Post-hoc comparisons revealed a significant up-regulation of ACR14 (169 ± 16%) and RUN14 (159 ± 19%) as compared to CTR14 (100 ± 4%; *p* < 0.05), (Figure 1B, 1C). Furthermore, a parallel trend towards an increase in the expression levels of PGC-1α was detected for both treatments after 7 days (F_(2, 21)_ = 3.11, *p* = 0.066). Significant elevations in PGC-1α levels were observed after 14 days (F_(2, 19)_ = 5.03, *p* < 0.02), with specific comparisons showing that ACR14 (183 ± 22%) and RUN14 (171 ± 16%) differed from CTR14 (100 ± 12%; *p* < 0.05), (Figure 1D, 1E). GLUT4 protein levels were augmented by AICAR at 7 days (F_(2, 21)_ = 3.73, *p* < 0.04). Post-hoc comparisons showed that ACR7 (134 ± 8%), but not RUN7 (117 ± 7%) differed significantly from CTR7 (100 ± 7%; *p* < 0.05). After 14 days, levels of GLUT4 are robustly up-regulated by both treatments (F_(2, 19)_ = 4.77, *p* < 0.02). Specific comparisons indicated that both ACR14 (173 ± 17%) and RUN14 (167 ± 16%) differ significantly from CTR14 (100 ± 13%; *p* < 0.05), (Figure 1F, 1G).

### AICAR and exercise elevate brain pAMPK

**Figure 2:**
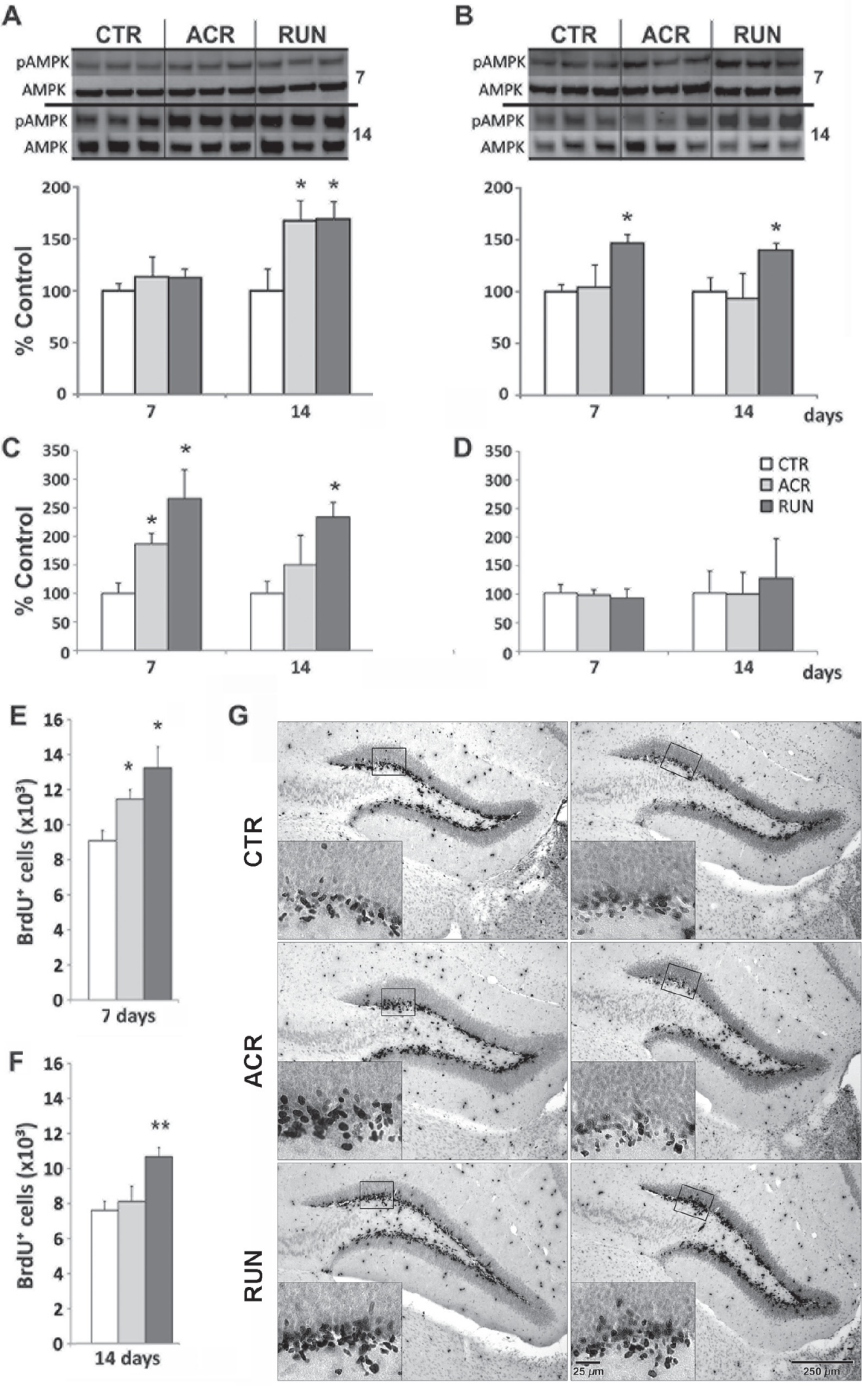
Effects of AICAR and running on AMPK activation and synaptic plasticity markers in dentate gyrus (DG) and lateral entorhinal cortex (LEC) **A, B.** AMPK phosphorylation (pAMPK) in (A) DG and (B) LEC, immunoblotting of tissue after 7 days and 14 days of treatment in control (CTR), AICAR treated (ACR) and voluntary running (RUN) mice. pAMPK is increased in (A) DG by both interventions at 14 days and (B) in the LEC only by running. **C.** DG BDNF levels are elevated in ACR7, RUN7 and RUN14 but not in the ACR14 group; **D.** No change in BDNF protein in the LEC; **E–F.** DG BrdU-positive cell number increases in ACR7, RUN7 and RUN14 but not ACR14 mice; **G.** Photomicrographs of BrdU-positive cells after 7 (left column) and 14 (right column) days. Sections derived from CTR7 and CTR14 groups (first row), ACR7 and ACR14 (second row) and RUN7 and RUN14 (third row) mice. Scale bar represents 250 μm in overview images and 25 μm in the high magnification inserts. (*p < 0.05; compared to CTR; **p < 0.05 compared to CTR and ACR). Error bars denote S.E.M.

To assess whether AICAR activates the AMPK pathway in the DG and LEC, we evaluated the expression levels of pAMPK at 7 and 14 days of treatment (*n* = 4–8 per group). One way ANOVA showed no significant increase in DG pAMPK levels at 7 days for both treatments: ACR7 (114 ± 20%) and RUN7 (112 ± 9%) as compared to CTR7 (100 ± 7%). However, a significant elevation was recorded at 14 days (F_(2, 13)_ = 7.682, *p* < 0.01), for both ACR14 (167 ± 19%) and RUN14 (168 ± 18%) as compared to CTR14 (100 ± 21%; *p* < 0.05). Interestingly, in the LEC only exercise increased pAMPK levels at both time points: (F_(2, 9)_ = 4.69, *p* < 0.04) RUN7 (147 ± 9%) as compared to CTR7 (100 ± 7%; *p* < 0.05) and (F_(2, 13)_ = 3.84, *p* < 0.05) RUN14 (140 ± 8%) as compared to CTR14 (100 ± 14%; *p* < 0.05), whereas neither ACR7 (102 ± 17%) nor ACR14 (94 ± 24%) differed from respective controls (Figure 2A–2B).

### AICAR and exercise regulate DG BDNF protein

Effects of both regimens on BDNF protein levels in the DG and LEC of animals treated for 7 days and 14 days were evaluated. One-way ANOVA showed a significant increase in DG BDNF levels after both AICAR and wheel running for 7 days (F_(2, 26)_ = 5.56, *p* = 0.0098). Specific comparisons showed that ACR7 (188 ± 26%) and RUN7 (265 ± 50%) differed from CTR7 (100 ± 12%; *p* < 0.05). After 14 days only exercise still showed a significant increase in DG BDNF levels (F_(2, 18)_ = 4.28, *p* < 0.031), as evidenced by a post-hoc comparison between RUN14 (223 ± 32%) and CTR14 (100 ± 22%; *p* < 0.05), whereas AICAR treatment did not, (Figure 2C). No significant increase in LEC BDNF levels was observed at these time points (Figure 2D).

### Dentate gyrus (DG) cell proliferation

The thymidine analog BrdU was injected daily for seven days prior sacrifice. BrdU positive cells were counted in the granule cell layer. One way ANOVA revealed a significant increase in cell proliferation at the 7-day time-point (F_(2, 21)_ = 5.26, *p* < 0.01). Post-hoc comparisons showed that ACR7 (11444 ± 533 cells; 126 ± 6%; *p* < 0.05) and RUN7 (13232 ± 1286 cells; 146 ± 14%) differed significantly from control, CTR7 (9092 ± 598 cells, 100 ± 7%; *p* < 0.05), (Figure 2E–2G). Furthermore, as observed for DG BDNF levels at the 14 day time-point, cell proliferation was only increased by running (F_(2, 19)_ = 8.40, *p* < 0.002). Indeed, RUN14 (10867 ± 368 cells; 143 ± 5%), but not ACR14 (7996 ± 874 cells; 105 ± 12%), differed significantly from CTR14 (7589 ± 508 cells; 100% ± 7%), (Figure 2F–2G).

### Differential effects of AICAR and exercise on oxidative stress and inflammation markers

**Figure 3:**
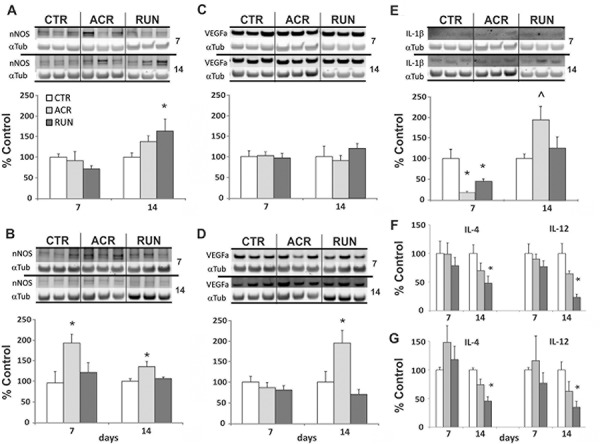
Expression levels of oxidative stress and inflammation markers in the brain **A.** DG and **B.** LEC nNOS immunoblotting of tissue after 7 and 14 days of treatment in control (CTR), AICAR treated (ACR) and voluntary running (RUN) mice. (A) DG nNOS is increased in by running after 14 days. (B) LEC nNOS is elevated by AICAR at every time point. **C.** DG and **D.** LEC VEGFa immunoblotting. (C) DG VEGFa levels are not increased. (D) LEC VEGFa increases after 14 days of AICAR **E.** DG IL-1β immunoblotting after 7 and 14 days; IL-1β levels are reduced by both treatments after 7 days but increased by AICAR after 14 days. (*p < 0.05; ^˄^*p* = 0.052). **F.** DG and **G.** LEC inflammation markers ELISA shows that 14 days of running decreases IL-4 and IL-12 levels. (*p < 0.05). Error bars denote S.E.M.

To assess whether divergent AICAR and exercise outcomes observed after fourteen days of treatment may be due to oxidative stress and or inflammation in the brain, we evaluated the expression levels of neuronal nitric oxide synthase (nNOS) as an oxidative stress marker, of interleukin-1β (IL-1β) as inflammatory marker and of vascular endothelial growth factor a (VEGFa) as a measure of vascularization. Protein levels were evaluated both in DG and in LEC at 7 and 14 days of treatment (*n* = 4–8 per group). No significant increase of nNOS was recorded in the DG after 7 days of treatment: ACR7 (91± 22%) and RUN7 (72 ± 9%) as compared to CTR7 (100 ± 8%). One way ANOVA showed divergent expression levels of DG nNOS after 14 days (F_(2, 13)_ = 3.814, *p* < 0.05) with a significant increase in RUN14 (159 ± 30%) but not in ACR14 (131 ± 16%). Interestingly, one way ANOVA showed that AICAR increased LEC nNOS levels both after 7 days (F_(2, 13)_ = 3.814, *p* < 0.05) and 14 days (F_(2, 13)_ = 5.269, *p* < 0.05). AICAR elevated LEC nNOS in ACR7 (200 ± 22%) as compared to RUN7 (126.3 ± 25%) and CTR7 (100 ± 28%), and in ACR14 (135 ± 12.1%) as compared to RUN14 (107 ± 3%) and CTR14 (100 ± 6%), (Figure 3A–3B).

VEGFa expression also differed between AICAR and exercise, albeit in LEC but not in DG. In the DG both at 7 days and 14 days VEGFa expression did not differ from CTR groups: ACR7 (101 ± 8%), RUN7 (97 ± 8%) CTR7 (100 ± 10%), and ACR14 (89 ± 12%), RUN14 (113 ± 8%) CTR14 (100 ± 3%). In the LEC VEGFa expression was significantly affected by AICAR after 14 days (F_(2, 13)_ = 4.625, *p* < 0.05). Specifically, VEGFa expression was increased in ACR14 (193 ± 30%), as compared to RUN14 (70 ± 11%) and CTR14 (100 ± 25%), whereas seven days of treatment had no effect: ACR7 (86.7 ± 12%), RUN7 (80.9 ± 11%), CTR7 (100 ± 14%) (Figure 3C–3D).

A remarkable decrease in DG IL-1β levels induced by both treatments was observed at 7days (F_(2.13)_ = 3.814, *p* < 0.05). Both ACR7 (22 ± 2%) and RUN7 (45 ± 8%) showed reduced levels of IL-1β as compared to CTR7 (100 ± 24%). An inversion of AICAR's effect, but not of exercise, was recorded at 14 days (F_(2, 13)_ = 3.712, *p* = 0.052). ACR14 (197.3 ± 48%) induced an increase in IL-1β levels, but not RUN14 (128.3 ± 29%) as compared to CTR14 (100 ± 11%). LEC levels of IL-1β were not detectable. (Figure 3E).

### Running decreases DG and LEC cytokines

Effects of running and AICAR on cytokine levels in the DG and LEC of animals treated for 7 days and 14 days (*n* = 3–4) was evaluated. There was no change in DG or LEC IL-4 and IL-12 levels with both treatments after 7 days. After 14 days both IL-4 and IL-12 levels were significantly affected in the DG (IL-4: F_(2, 9)_ = 4.33, *p* < 0.05; IL-12: F_(2, 9)_ = 4.78, *p* < 0.05). Specific comparisons evidenced that RUN14 levels of IL-4 (48 ± 12%) and IL-12 (23 ± 5%) were lower than that of controls: CTR14, DG IL-4 (100 ± 15%) and DG IL-12 (100 ± 29%) (Figure 3F). Similarly, one way ANOVA showed significant reductions in the running group of LEC IL-4 (F_(2, 6)_ = 13.5, *p* < 0.01) and LEC IL-12 levels (F_(2, 6)_ = 5.52, *p* < 0.05). Post hoc analysis showed LEC RUN14 levels of IL-4 (45 ± 8%) and IL-12 (35 ± 10%) differed from CTR14 IL-4 (100 ± 4%) and IL-12 (100 ± 14%) levels (Figure 3G).

### Regulation of gene expression by AICAR and exercise

Microarray analysis of DG and LEC tissue derived from animals in control, AICAR treated and running groups was performed after 7 and 14 days of treatment. Interestingly, the array results were consistent with the transiently beneficial effects of AICAR treatment on the brain. Parallel effects of exercise and AICAR on DG gene expression were observed with 7 days, but not 14 days of AICAR treatment.

### Dentate gyrus gene expression profile

**Figure 4:**
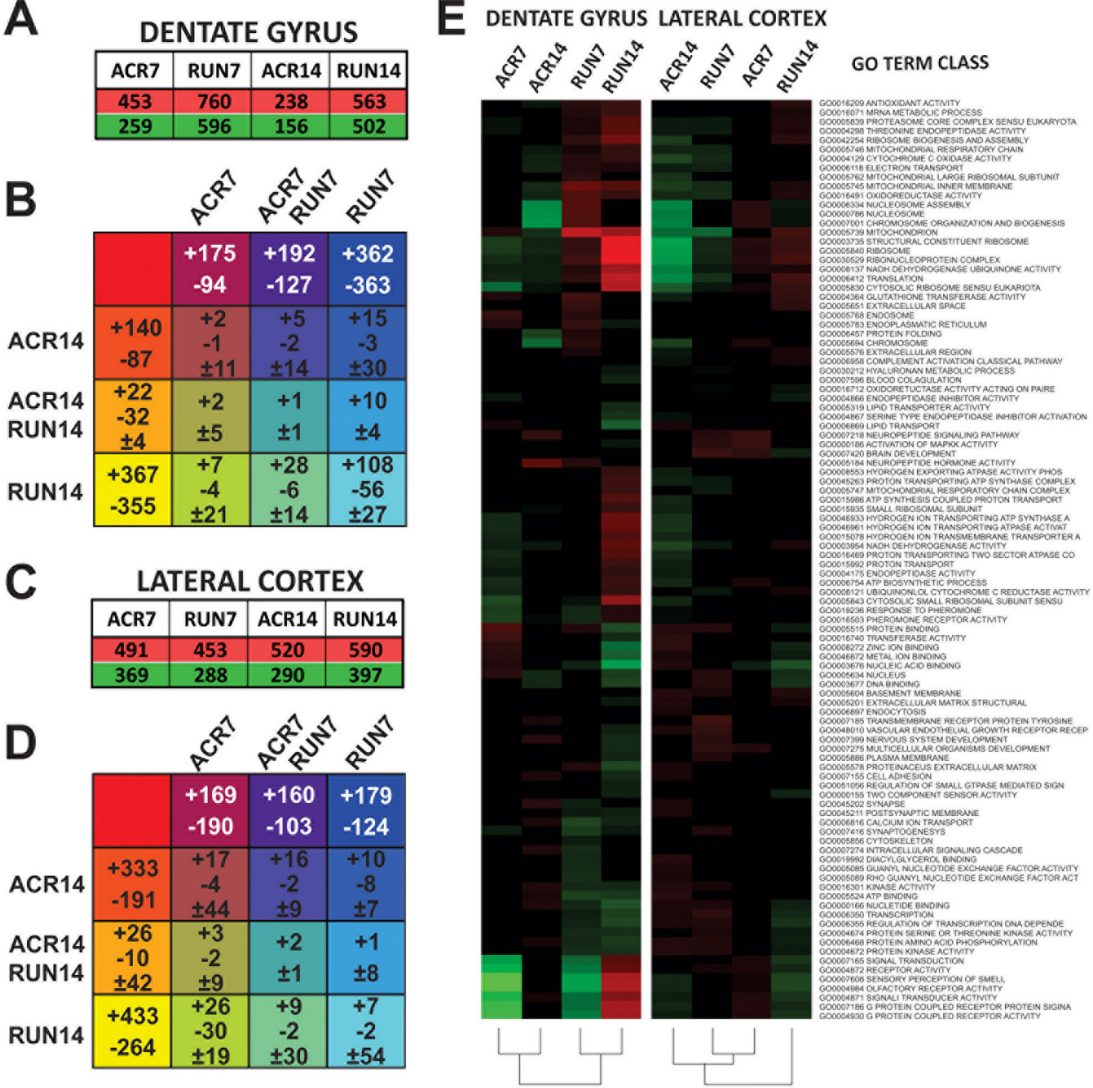
Microarray analysis of dentate gyrus (DG) and lateral entorhinal cortex (LEC): analysis of control (CTR), AICAR (ACR), and voluntary running (RUN) mice after 7 and 14 days of treatment **A.** Total up- and down-regulated genes per treatment group in the DG. **B.** Karnaugh map reporting overlap of DG gene regulation by all treatment groups. + values represent upregulated genes, while - values represent down-regulated genes and ± values represent genes with different regulation within groups. **C.** Total up- and down-regulated genes per treatment group in the LEC. **D.** Karnaugh map reporting overlap of LEC gene regulation by all treatment groups. **E.** Heat map of GO Term gene classes. Up-regulated classes are colored in red, down-regulated gene classes are colored in green.

Microarray analysis of DG tissue showed a considerable number of genes up- and down-regulated by both treatments; 7 days of AICAR administration up-regulated 453 genes and down-regulated 259, while 14 days of administration up-regulated 238 and down-regulated 156. Interestingly, exercise affected more genes than drug administration, increasing expression of 760 genes and decreasing 596 genes after 7 days, and increasing expression of 563 genes and reducing 502 genes after 14 days (Figure 4A).

In order to study similarities of gene regulation between groups, the overlap of affected genes was analyzed by comparing all groups. Specifically, we compiled a Truth table and represented the overlapping results in a Karnaugh map. Notably, the overlap between 7 days of AICAR (ACR7) and running (RUN7) was strikingly higher (parallel up-regulation of 192 genes and down-regulation of 147 genes), than between 14 days of AICAR (ACR14) and running (RUN14), (only 22 genes are upregulated and 32 down-regulated in a parallel fashion), (Figure 4B).

GO Term gene classes significance was reported with a selector value equals 2 or −2 (*z*-ratio ≥ 1.5 or ≤ −1.5, *P*-value ≤ 0.05, fdr ≤ 0.3, and average signal intensity > 0). Overall results of all GO Term classes showed differences between running and drug administration (Figure 4E). Such divergence is maintained when analysis is restricted to cellular components and molecular functions GO Term classes (Supplementary Figure S1B), while a more parallel effect between short-time running and AICAR emerges in biological processes (Supplementary Figure S1A and Figure S1C). We therefore restricted our analysis to classes more relevant to brain function and metabolic activity. Interestingly, results showed a remarkable effect on neuron-related gene classes in the DG: genes important for synaptic transmission and neuropeptide signaling are down-regulated by both exercise and short term AICAR administration. Seven days of treatment showed pronounced overlap of up- and down-regulated genes, with 361 genes similarly affected by AICAR and exercise in the DG. Of these selected genes (*z*-ratio ≥ 1.5 or ≤ −1.5, *P*-value ≤ 0.05, fdr ≤ 0.3, and average signal intensity > 0), 104 genes in DG are known to directly affect neuronal plasticity and survival.

**Figure 5:**
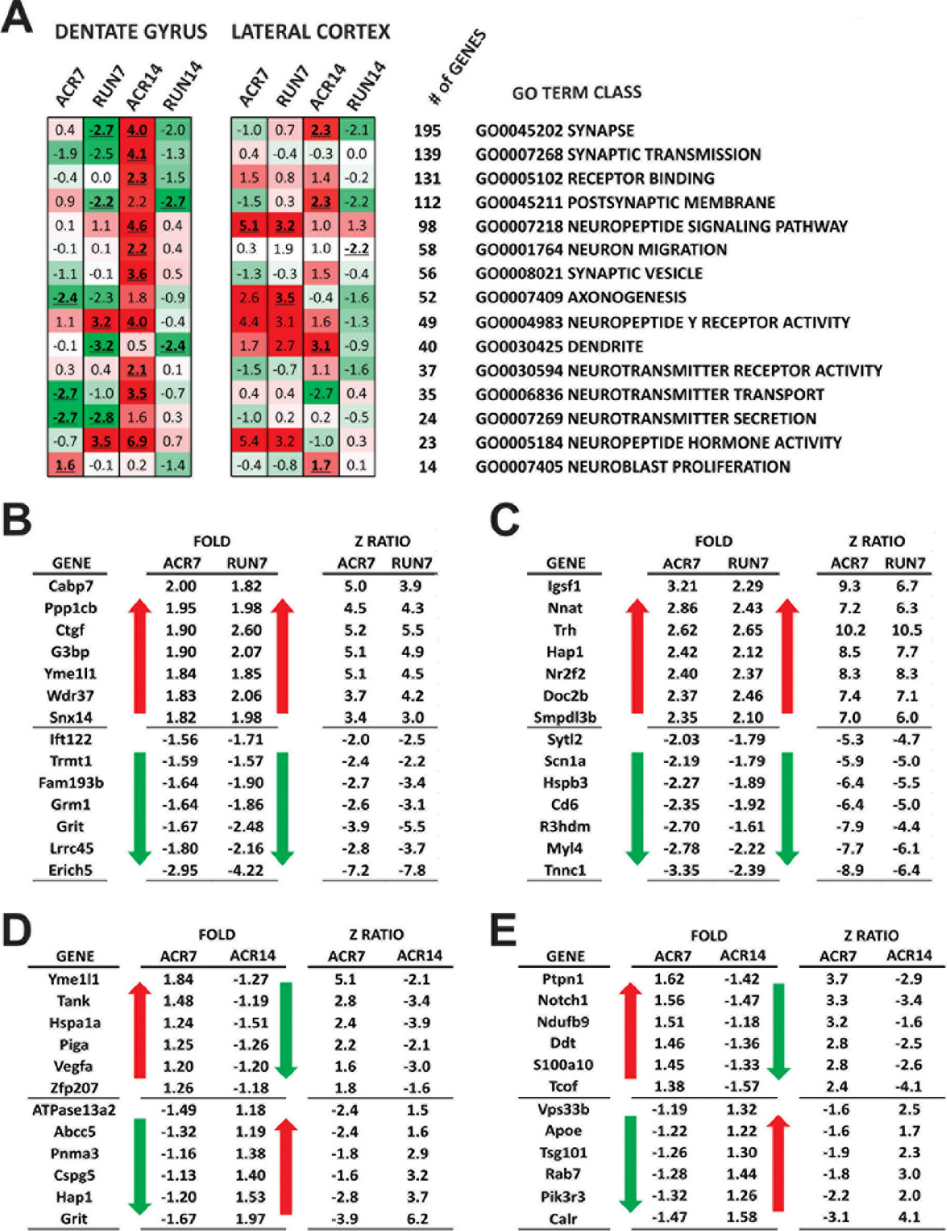
Microarray analysis of dentate gyrus (DG) and lateral entorhinal cortex (LEC): neuronal gene classes **A.** Heat map represents the expression of selected neuro-related GO Term gene classes. Up-regulated classes are colored in red, down-regulated classes are colored in green. For every class the *Z*-ratio value is reported. Bold, underlined *Z*-ratio values represent classes with a Selector value above 2 or below −2. **B–C.** Top regulated genes with parallel regulation after 7 days of AICAR and running regimen. The tables report the most up- and down-regulated genes for (B) DG and (C) LEC; the red arrow marks up-regulation, the green arrow down-regulation; for each gene Fold of Increase and *Z*-Ratio are reported; **D–E.** Top regulated genes with opposite regulation between 7 days and 14 days of AICAR. The tables report the most up- and down-regulated genes for (D) DG and (E) LEC; the red arrow marks up-regulation, the green arrow down-regulation; for each gene Fold of Increase and Z-Ratio are reported.

A striking difference between AICAR and exercise appears after 14 days of treatment: with longer running duration (RUN14), the examined GO Term neuro-related classes maintain or even increase the down-regulation observed after 7 days of treatment, while 14 days of AICAR resulted in a prominent up-regulation in the DG (Figure 5A). The inversion of gene regulation between 7 and 14 days of AICAR led us to select 31 genes in DG that shifted from up- to down-regulation and vice-versa (Figure 5D). In the DG, 16 of the selected genes are known to be involved in neuronal plasticity processes; e.g. *Grit* (a.k.a. *p250GAP*) (ACR7 = −1.67 fold, ACR14 = 1.97 fold), which regulates axon growth [28] and whose up-regulation was shown to inhibit BDNF-induced axonal branching [29]; *Hap1* (ACR7 = −1.20 fold, ACR14 = 1.53 fold). *Grit* is involved in regulation of neuronal mTORC1 signaling and neuronal morphogenesis [30] and plays a crucial role in postnatal neurogenesis and neurotrophin receptor sorting [31]. In addition, *Hspa1a* (a.k.a. *Hsp72*) (ACR7 = 1.24 fold, ACR14 = −1.51 fold), identified as an oxidative stress-neuroprotective-astrocyte activator [32], *ATPase132a* (ACR7 = −1.49 fold, ACR14 = 1.18 fold), previously shown to regulate neuronal integrity, intracellular cation flux and mitochondrial morphology [33], as well as *Yme1l1* (ACR7 = 1.84 fold, ACR14 = −1.27 fold), involved in maintaining mitochondrial functions [34].

**Figure 6:**
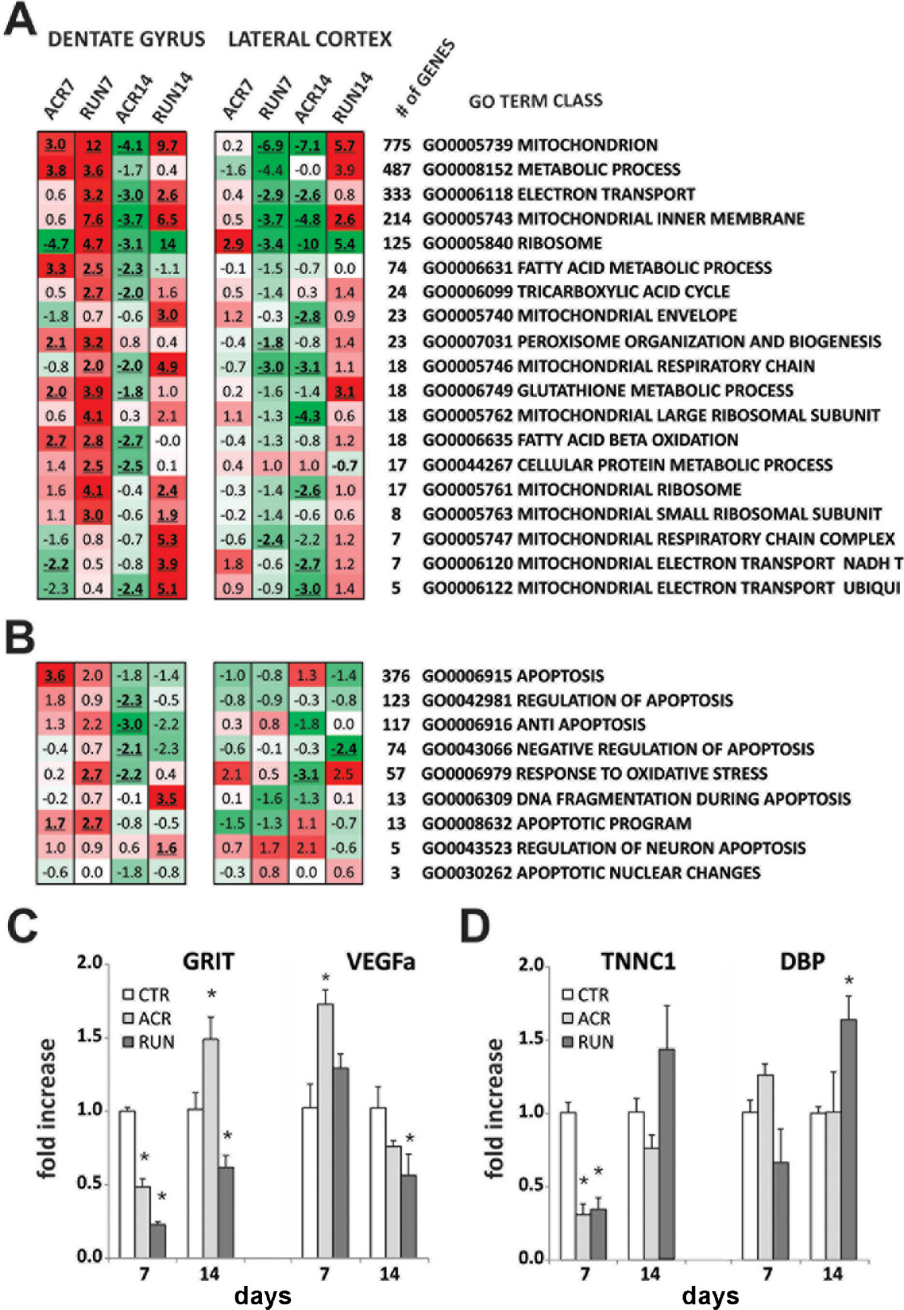
Microarray analysis of dentate gyrus (DG) and lateral entorhinal cortex (LEC): energy metabolism gene classes **A.** Heat map represents the expression of selected mitochondrial- and energy-related GO Term gene classes. Up-regulated classes are colored in red, down-regulated classes are colored in green. For every class the *Z*-ratio value is reported. Bold, underlined *Z*-ratio values represent classes with a Selector value above 2 or below −2. **B.** Heat map represents the expression of selected cell death- and apoptosis-related GO Term gene classes. Up-regulated classes are colored in red, down-regulated classes are colored in green. For every class the *Z*-ratio value is reported. Bold, underlined *Z*-ratio values represent classes with a Selector value above 2 or below −2. Relative gene expression levels in **C–D.** CTR, ACR and RUN mice; RT-qPCR analysis of expression levels of two genes in (C) DG and two genes in (D) LEC at 7 and 14 days of treatment (*p < 0.05). Error bars indicate S.E.M.

We also analyzed energy- and mitochondrial-related GO Term gene classes: our study showed effects of both treatments on DG gene regulation; classes such as mitochondrion, metabolic process, mitochondrial inner membrane, fatty acid and glutathione metabolic processes, are up-regulated at each time point of the exercise regimen and by short term (7 days) AICAR administration. Once again, a striking inversion of gene regulation appeared after 14 days of drug treatment, with a remarkable down-regulation of the analyzed classes (Figure 6A).

Moreover, in view of the lack of increase in hippocampal proliferation after 14 days of AICAR administration, we evaluated apoptosis- and cell death-related GO Term gene classes. The results showed an up-regulation for gene classes that enhance apoptosis (activation or increase of frequency, rate or extent of cell death by apoptotic process) and a substantial down-regulation for gene classes that decrease apoptotic processes (Figure 6B).

### Lateral entorhinal cortex gene expression profile

Exercise and AICAR treatment modified the LEC gene expression profile, albeit with a different pattern than for the DG. Seven days of AICAR administration up-regulated 491 genes and down-regulated 369, while 14 days of administration up-regulated 520 and down-regulated 290. In contrast to the DG, exercise and AICAR affected LEC more equally, with running increasing expression of 453 genes and decreasing 288 genes after 7 days, and increasing expression of 590 genes and reducing 397 genes after 14 days (Figure 4C). The overlap of affected genes was analyzed by comparing all groups. The resulting Karnaugh map is reported in Figure 4D. Similar to gene regulation in the DG, the overlap between 7 days of AICAR and running was strikingly higher (parallel up-regulation of 160 gene and down-regulation of 103 genes) than between 14 days of AICAR and running (only 26 genes are up-regulated and 10 down-regulated in a parallel fashion).

The overall comparison of all GO Term classes in the LEC showed that treatment duration is the main factor for class profile similarity: short-term treatment of both AICAR and exercise are more similar to each other than to their respective long-term group (Figure 4E). Such parallel regulation is maintained when analysis is restricted to the three GO Term class subgroups, biological processes, cellular components and molecular functions (Supplementary Figure S1A–S1B–S1C). More specifically, 14 days of AICAR up-regulated neuro-related gene classes, similar to the pattern observed in the DG of the ACR14 group (Figure 5A). Furthermore, as observed in the DG, longer running (14 days) down-regulated neuro-related gene classes.

For further evaluation we selected LEC genes that switched from up- to down-regulation between short (ACR7) and long (ACR14) drug treatment (Figure 5E). Of the 59 selected genes 18 are known to be involved in neuronal survival and plasticity processes. For example, *Calr* (ACR7 = −1.47 fold, ACR14 = 1.58 fold), affects Ca^+^ influx and is linked with neurogenesis as well as neuronal development [35]. In addition, the genes *Ddt* (ACR7 = 1.46 fold, ACR14 = −1.36 fold) a possible neurotrophic factor [36]; *Ptpn1* (ACR7 = 1.62 fold, ACR14 = −1.42 fold), which upon brain deficiency induces hypothalamic impairment of AMPK activation [37]; *Notch1* (ACR7 = 1.56 fold, ACR14 = −1.47 fold), important for postnatal neurogenesis, dendritic morphology [38], and synaptic plasticity [39]; and *Rab7* (ACR7 = 1.28 fold, ACR14 = −1.44 fold) when down-regulated affects axonal transport and diminishes NGF retrograde signaling [40].

Analysis of gene classes important for regulation of energy metabolism and mitochondrial function in LEC showed a strong down-regulation in the RUN7 group, while short drug administration (ACR7) only mildly modified LEC gene expression. At the longer, 14-day time point, however, a striking difference between the two treatments emerged: exercise promoted an intense up-regulation of gene expression, while ACR14 resulted in a remarkable down-regulation (Figure 6A).

### Novel target genes regulated by AICAR and running

The microarray data also revealed genes so far not known to play major roles in neuronal plasticity and cognitive function, but that are nonetheless regulated in a similar fashion and intensity to neuro-related genes.

### Novel target genes in dentate gyrus

In the DG the groups ACR7 and RUN7 induced similar regulation of 361 genes, of which only 104 are known to be involved in neuronal plasticity and survival. Among the top 5 up-regulated novel genes (Table 2), we report *Wdr37* (ACR7 = 1.83 fold, RUN7 = 2.06 fold), *Armc8* (ACR7 = 1.79 fold, RUN7 = 1.98 fold), and *Phactr1* (ACR7 = 1.77 fold, RUN7 = 1.71 fold), so far known respectively for their role in kidney function and chronic kidney disease [41]; carcinogenesis [42]; and for promoting capillary tube formation in endothelial cells [43]. Among the down-regulated genes, the microarray analysis highlighted *Erich5* (ACR7 = −2.95 fold, RUN7 = −4.42 fold), *Lrrc45* (ACR7 = −1.80 fold, RUN7 = −2.16 fold) and *Fam193b* (ACR7 = −1.64 fold, RUN7 = −1.90 fold), whose known functions so far are respectively unidentified, related to centrosome cohesion [44], and to rhabdomyosarcomagenesis [45].

**Table 2:** List of top 5 up- and down-regulated, novel candidate genes in Dentate Gyrus and Lateral Entorhinal Cortex after 7 days of AICAR administration (ACR7) and exercise (RUN7)

DENTATE GYRUS					LATERAL ENTORHINAL CORTEX				
GENE	FOLD		Z RATIO		GENE	FOLD		Z RATIO	
	ACR7	RUN7	ACR7	RUN7		ACR7	RUN7	ACR7	RUN7
Wdr37	1.83	2.06	3.73	4.21	Igsf1	3.21	2.29	9.34	6.72
Armc8	1.79	1.98	4.67	4.65	Nr2f2	2.40	2.37	8.29	8.33
Phactr1	1.77	1.71	1.46	0.79	Smpdl3b	2.35	2.10	6.96	5.97
Zmpste24	1.76	1.70	3.92	3.08	Tmie	2.19	1.93	6.27	5.28
Pfkp	1.67	2.23	2.65	3.03	Gm4983	1.90	1.96	5.95	6.45
Ift122	−1.56	−1.71	−2.04	−2.53	Scn1a	−2.19	−1.79	−5.89	−5.02
Trmt1	−1.59	−1.57	−2.44	−2.23	Cd6	−2.35	−1.92	−6.35	−4.97
Fam193b	−1.64	−1.90	−2.74	−3.35	R3 hdm	−2.70	−1.61	−7.89	−4.40
Lrrc45	−1.80	−2.16	−2.80	−3.71	Myl4	−2.78	−2.22	−7.65	−6.05
Erich5	−2.95	−4.22	−7.20	−7.82	Tnnc1	−3.35	−2.39	−8.91	−6.42

In the DG, of the reported 31 genes that showed an inversion of regulation between 7 and 14 days of AICAR treatment, 15 are not related to neuronal plasticity and survival. The top 5 novel gene candidates are reported in Table 3. In the DG, among the genes that showed up-regulation after 7 days and down-regulation after 14 days of AICAR, we report for example, *Lurap1l* (ACR7 = 1.78 fold, ACR14 = −1.32 fold), *Ube2g1* (ACR7 = 1.48 fold, ACR14 = −1.26 fold) and *Tank* (ACR7 = 1.48 fold, ACR14 = −1.19 fold), whose gene functions are respectively unidentified, related to the efficiency of the ubiquitin-proteasome protein degradation system [46], and related to osteoclastogenesys and differentiation [47]. Furthermore, genes down-regulated after 7 days and up-regulated after 14 days of AICAR include *Cacna2d1* (ACR7 = −1.42 fold, ACR14 = 1.34 fold), *Slc4a3* (ACR7 = −1.41 fold, ACR14= 1.28 fold) and *Tro* (ACR7 = −1.37 fold, ACR14= 1.20 fold), genes known to play a role in the regulation of calcium channel complex [48], cell volume, pH and Cl^−^ levels [49] and in sperm motility [50] (Table 3).

**Table 3:** List of top 5 up- and down-regulated, novel candidate genes in Dentate Gyrus and Lateral Entorhinal Cortex after 7 days (ACR7) and 14 days (ACR14) of AICAR administration

DENTATE GYRUS					LATERAL ENTORHINAL CORTEX				
GENE	FOLD		Z RATIO		GENE	FOLD		Z RATIO	
	ACR7	ACR14	ACR7	ACR14		ACR7	ACR14	ACR7	ACR14
Lurap1l	1.78	−1.32	3.38	−2.68	Pla2g7	1.69	−1.38	3.97	−2.81
Ube2g1	1.48	−1.26	2.38	−2.20	Kti12	1.64	−1.23	3.79	−1.82
Tank	1.48	−1.19	2.82	−1.63	Ube2q	1.54	−1.16	3.49	−1.57
Jundm2	1.47	−1.43	3.10	−3.36	Eif3k	1.50	−1.29	3.23	−2.44
Kansl1l	1.35	−1.33	2.65	−2.69	Atp5e	1.43	−1.40	2.64	−2.77
Abcc5	−1.32	1.19	−2.39	1.61	Gpcpd1	−1.42	1.45	−2.88	3.21
B4galnt4	−1.34	1.26	−1.53	2.22	Zfand6	−1.43	1.29	−2.67	2.19
Tro	−1.37	1.20	−2.23	1.48	Eif4a2	−1.46	1.36	−2.55	2.18
Slc4a3	−1.41	1.28	−2.40	2.26	Fam126b	−1.48	1.21	−3.27	1.67
Cacna2d1	−1.42	1.34	−3.55	2.64	AI593442	−1.82	1.20	−4.71	1.58

### Novel target genes in lateral entorhinal cortex

In the LEC, ACR7 and RUN7 groups showed similar regulation in of 294 genes, of which only 78 are known to be involved in neuronal plasticity and survival. The top 5 novel LEC genes, similarly up- and down-regulated after 7 days, are reported in Table 2. Among them, we report, for instance, *Igsf1* (ACR7 = 3.21 fold, RUN7 = 2.29 fold), *Nr2f2* (ACR7 = 2.40 fold, RUN7 = 2.37 fold) and *Smpdl3b* (ACR7 = 2.35 fold, RUN7 = 2.10 fold), whose known functions are so far related to membrane trafficking [51], embryonic organ development [52], and cytoskeleton remodeling in podocytes [53]. Among the down-regulated LEC candidates we report *Tnnc1* (ACR7 = −3.35 fold, RUN7 = −2.39 fold), *Myl4* (ACR7 = −2.78 fold, RUN7 = −2.22 fold) and *R3 hdm* (ACR7 = −2.70 fold, RUN7 = −1.61 fold), whose functions are respectively related to cardiac myofilaments contraction [54], to myosin contraction in mice [55], and still unidentified within the mouse genome.

Of the 59 genes in LEC, that showed an inversion of regulation between 7 and 14 days of AICAR treatment, 41 are not related to neuronal plasticity and survival. Indeed, the top 5 LEC candidates include, for instance, *Pla2g7* (ACR7 = 1.69 fold, ACR14 = −1.38 fold), *Kti12* (ACR7 = 1.64 fold, ACR14 = −1.23 fold) and Ube2q (ACR7 = 1.54 fold, ACR14 = −1.16 fold), whose known functions are respectively related to smooth muscle differentiation and influenza susceptibility [56, 57]; to still unidentified processes in mice; and to female fertility [58]. Finally, genes *AI593442* (ACR7 = −1.82 fold, ACR14 = 1.20 fold), *Fam126b* (ACR7 = −1.48 fold, ACR14= 1.21 fold) and *Eif4a2* (ACR7 = −1.46 fold, ACR14= 1.36 fold), are respectively still unknown; unidentified; and play a role in breast, lung and skin cancer [59] (Table 3).

### RT-qPCR validates microarray results

The qPCR results were consistent with the microarray findings. Four relevant genes were selected for qPCR validation of microarray results (*n* = 4). All chosen genes presented a selector value equals 2 or −2 (*z*-ratio ≥ 1.5 or ≤ −1.5, *p* ≤ 0.05, fdr ≤ 0.3, and average signal intensity > 0). RT-qPCR was conducted for *Grit* and *VegfA* (DG) and *Tnnc1* and *Dbp* (LEC) at 7 and 14 days of treatment. Specifically, one way ANOVA and post-hoc analysis showed that *Grit* was significantly down-regulated in DG at 7 days (F_(2, 6)_ = 115.0, *p* < 0.001) for both treatments (ACR7 = 0.49 ± 0.05 fold, *p* < 0.05; RUN7 = 0.23 ± 0.02 fold, *p* < 0.05) and at 14 days (F_(2, 11)_ = 16.4, *p* < 0.001; ACR14 = 1.46 ± 0.12, *p* < 0.05; RUN14 = 0.64 ± 0.09, *p* < 0.05). Moreover, *VegfA* was up-regulated by AICAR after 7 days (F_(2, 6)_ = 7.377, *p* < 0.024; ACR7 = 1.73 ± 0.10 fold, *p* < 0.05), and significantly down-regulated by running after 14 days (F_(2, 11)_ = 4.808, *p* < 0.032; RUN14 = 0.60 ± 0.04 fold, *p* < 0.05) (Figure 6C). *Tnnc1* regulation in the LEC showed significant down-regulation after 7 days of both treatment (F_(2, 9)_ = 6.004, *p* < 0.23; ACR7 = 0.30 ± 0.06 fold, *p* < 0.05; RUN7 = 0.36 ± 0.09 fold, *p* < 0.05); lastly, *Dbp* was upregulated by exercise after 14 days (F_(2, 11)_ = 5.19, *p* < 0.03; RUN14 = 1.76 ± 0.18 fold, *p* < 0.05), (Figure 6D).

## DISCUSSION

Our study demonstrates that voluntary exercise and AICAR administration similarly activate muscle metabolism. Initial elevations of muscle AMPK were observed after seven days and robust up-regulation occurred after fourteen days of interventions. In the brain, seven days of AICAR or running increased dentate gyrus BDNF protein levels and cell proliferation. However, longer pharmacological activation did not result in changes in cell genesis or neurotrophin levels and may even be detrimental. In particular, microarray analysis showed an inversion of DG gene regulation, such as increased expression of pro-apoptotic genes, with long-term AICAR treatment. In addition, markers of inflammation were up-regulated in the DG and LEC after fourteen days of AICAR treatment, whereas running reduced inflammatory cytokine levels. Thus, while both interventions may have similar effects on muscle energy metabolism, only running continuously benefits brain function.

AMPK activation in muscle is an important factor in regulating mitochondrial proteins and exercise endurance training [60]. We made side-by-side comparisons *in vivo* over time to determine effects of exercise and AICAR on multiple components of the energy-sensing network in muscle, including pAMPK, PGC-1α and GLUT4. Our data show that all three components were up-regulated in muscle to a similar extent by AICAR and exercise after 14 days of treatment. In human and rodent skeletal muscle, pAMPK levels increase acutely after a bout of exercise [61] and after 15, 30, and 60 minutes or 48 hours of brief AICAR administration [62]. Previous studies on chronic exercise training for 12 weeks in rodents also showed a notable increase in basal levels of pAMPK in peripheral tissues, such as skeletal muscle [20], liver and adipose tissue [63]. In addition, training up-regulates PGC-1α and overexpression of PGC-1α in muscle increases exercise performance [64]. Indeed, the effect of AMPK activation on PGC-1α is considered an important factor in regulating exercise training-induced adaptations, and may also mediate the AMPK-induced elevation in mRNA levels of GLUT4. This is supported by research showing that activation of AMPK in PGC-1α knock-out mice does not induce GLUT4 expression [65]. It should be noted that GLUT4 increased more slowly over time with running than by compound administration, suggesting that despite similar effects on the primary targets (pAMPK and PGC-1α), AICAR and exercise may differentially activate downstream signaling pathways. Indeed, more detailed analysis of both interventions in muscle may further understanding of their influence on brain function.

Several recent studies support the concept that muscle mediated signaling factors may influence brain plasticity. Administration of AMPK agonist AICAR enhances endurance in sedentary mice and functions as an ‘exercise-mimetic’ [20], improving adult neurogenesis and memory function [21]. The effects of the compound are likely indirect as AICAR has a low ability to cross the blood brain barrier [66]. Upon intracerebral infusion memory function and long-term potentiation in hippocampal slices are reduced [67]. In addition, AICAR does not improve spatial memory in mice selectively lacking functional AMPK in muscle, suggesting an indirect mechanism of action and a link between muscle and brain [22]. Further support for the idea that muscle energy metabolism affects brain function comes from a recent study showing that over-expression of PGC-1α in mouse muscle affects the kynurenine pathway and protects these mice from stress induced reduction of synaptic plasticity proteins in the brain, as well as from exhibiting depression-like behaviors [23, 68]. Overexpression of PGC-1α in muscle also has been reported to lead to increased production of Fibronectin type III domain containing 5 (FNDC5), a myokine that is released during exercise [69]. Enzymatic cleavage of FNDC5 generates a peptide called irisin, which may enter the brain and induce hippocampal BDNF gene expression [70].

In the brain, AMPK protein levels were up-regulated in the DG by exercise and AICAR to a similar extent as in muscle at the longer time-point (14 days), but not earlier (7 days). Consistently, thirty days of running elevates hippocampal PGC-1α levels, and eight weeks of treadmill training elevates PGC-1α and Sirtuin 1, increasing brain mitochondrial biogenesis [70]. In addition, eight weeks of voluntary exercise increased phosphorylation of AMPK in the hippocampus of senescence-accelerated mice [71]. These changes may mediate synaptic plasticity and spine formation [70, 72]. However, we observe that the beneficial effects of AICAR and exercise on the brain (increased DG cell genesis and BDNF levels at 7 days) precede brain energy metabolism protein level changes (at 14 days in DG and LEC), indicating these may be unnecessary for enhancement of neural plasticity. Indeed, in Alzheimer's Disease mouse models, prolonged brain AMPK activation may contribute to detrimental effects on synaptic plasticity and memory formation, by inducing long-lasting cellular stress and impairing protein synthesis [73].

A novel observation in the present study is that AICAR treatment elevates BDNF protein levels. However the increase was observed only after 7 days of AICAR treatment, whereas exercise consistently elevated BDNF DG protein levels. It should be noted that our microarray data did not show an exercise-induced increase in BDNF mRNA in the DG at these time-points, consistent with several other short-term [74] or short-distance studies [75], but not other running research [76, 77] analyzing whole hippocampus. Longer running paradigms elevate DG BDNF mRNA levels [78]. In the LEC, no increase in BDNF protein levels was found at 7 and 14 days with either intervention, and a reduction in BDNF mRNA was observed. Longer term running, however, elevates BDNF protein levels in the adjacent perirhinal cortex [8]. Overall, there appears to be dissociation between the effects of the interventions on BDNF protein levels and gene expression at the different time-points and in both brain regions examined. These discrepancies may be due in part to transport of BDNF from other brain regions [79].

The effects of both treatments were evaluated on DG and LEC protein expression of the oxidative stress marker nNOS. Running increases nNOS in the DG (after 14 days) but not in the LEC. Treatment with AICAR, on the other hand, does not increase expression in the DG but elevates nNOS levels in the LEC at both time points. This differential modulation, depending both on treatment length and brain region, led us to hypothesize that oxidative stress modulators may be, at least in part, responsible for the lack of improvement of brain functions after longer AICAR treatment. Both nitric oxide (NO) and nNOS affect neurogenesis and neuronal differentiation *in vitro* [80] and *in vivo* [81]. Furthermore, modulation of nNOS in rodents was shown to affect the rate of neurogenesis in the DG [82]. Moreover, glucose-inhibited neurons undergo an increase in nNOS activity and a reduction of pAMPK upon increasing glucose concentrations [83]; this correlation between nNOS increase and kinase reduction is similar to our findings on LEC protein levels. AICAR increases LEC nNOS levels without promoting AMPK activation, while running activates AMPK with no increase in nNOS. Further analyses of the modulation of NO by these treatments may clarify possible direct and indirect mechanisms of action.

In previous studies a wide range of doses and duration of administration of AICAR showed beneficial effects of this compound on peripheral processes. Lower AICAR concentrations than used in our study (0.25 mg/kg/day for two weeks) in mice improved vascular endothelial dysfunction [84]. In addition, administration of 8 mg/kg/min AICAR for 2 hours significantly inhibited glucose production in rodents [85]. We utilized the 500 mg/kg dose because previous studies showed enhanced endurance [20] and memory function [21]. In addition, longer durations of treatment than in our study (4 weeks [20] and 7 weeks [86], 500 mg/kg/day) and higher dosage (1g/kg [87, 88]) had beneficial effects on endurance [20], glucose tolerance and systolic blood pressure [86], myokine release [87] and skeletal muscle vascularization factors levels [88]. In the brain, however, we observed an increase in pro-inflammatory cytokines and angiogenic factors, namely an increase of IL-1β levels in the DG and an up-regulation of VEGFa in the LEC after 14 days of AICAR treatment. Pro-inflammatory molecules are known to affect angiogenesis. VEGFa and other angiogenic molecules can affect inflammation in a plethora of ways [89]. Specifically, cytokines like IL-1β and associated angiogenic modulators are reported to be a crucial component of brain and systemic degeneration processes in aging [90, 91] and in degenerative diseases [92, 93, 94], such as conversion from Mild Cognitive Impairment into Alzheimer's disease (for a review see [95]). Conversely, exercise is known to reduce inflammatory processes in the brain [6, 7, 96]. Consistently, we observed reduced levels of inflammatory cytokines IL-1β, IL-4 and IL-12 with running.

To further elucidate the mechanisms underlying the similarities and differences between AICAR and exercise treatment in the DG and LEC, we performed microarray analysis that matched the time-points of the cellular data. Gene regulation was consistent with plasticity results, showing a parallel regulation of neuro- and energy-related genes at short time points. An inversion of gene expression occurred upon prolonged pharmacological treatment. The neuro-related gene classes are down-regulated at short time points (ACR7 and RUN7) in the DG, and continue to be down-regulated at the longer time point (RUN14). However, a striking up-regulation appears when AICAR administration is extended (ACR14), including elevation of expression of genes important for apoptosis. In a parallel although different way, LEC gene neuro-related classes showed up-regulation at short time points (ACR7 and RUN7), but, as observed for the DG, gene regulation switches to remarkable down-regulation after longer training (RUN14). Longer pharmacological treatment with AICAR (14 days), however, prevented the onset of the down-regulation and maintained LEC gene classes up-regulated.

Our microarray data indicate that exercise and AICAR have a remarkable effect on gene regulation in DG and LEC. There are substantial differences between the two brain regions depending on the duration of the treatment and on the gene classes considered. In the LEC genes related to energy and mitochondrial regulation were modified by the interventions, whereas in the DG expression of neuro-related genes was altered. These data show that external stimuli, such as exercise and AICAR administration, target different functions in different brain areas in specific ways. Indeed, many of the running studies microarray analyses in rodents have been focused on the hippocampus [76, 97]. Regionally-specific differences are of interest, in the light of recent studies on gene profiles of various human brain regions. Such studies reported marked regional differences with aging in gene profiles of entorhinal cortex and hippocampus, with respect to other brain regions [98]. In addition, within the human brain metabolic differences between cortex (high glucose consumption) and other brain regions have been reported [99], which may be linked directly to synaptic plasticity.

Exercise-mimetics may be a promising alternative to physical activity in promoting brain function in aging or neurodegenerative diseases. However, muscle AMPK pathway activation may not predict central effects of such interventions. Even though exercise and AICAR resulted in similar muscle changes, in the brain differential patterns of responsiveness to drug administration developed over time. AICAR switched from being comparable to exercise to upregulating markers of apoptosis and inflammation. Thus, development of pharmacological agents that can consistently and safely mimic effects of exercise on the brain may prove to be challenging.

## MATERIALS AND METHODS

### Subjects and procedures

A cohort of 82 C75BL/6J male mice was purchased from the Jackson laboratory (Bar Harbor, ME). The one-month-old mice were individually housed in standard conditions with food and water ad libitum. Animals were divided into 3 groups [control (CTR), AICAR treated (ACR), voluntary running (RUN)] for experiments of 3, 7 or 14 days duration. ACR mice received a daily intraperitoneal injection (IP) of 5-aminoimidazole-4-carboxamide-1-β-D-ribofuranoside (AICAR, Toronto Research Chemicals Inc., Canada) of 500 mg/kg/day, dissolved in saline, similar to previous studies [20, 21, 22]; CTR and RUN animals received IP saline vehicle. RUN animals had free access to running wheels. Running distance was recorded using Clocklab software (Actimetrics, Wilmette, IL). A subset of the mice (*n* = 46) was treated in the same conditions (CTR, ACR, RUN), but also received daily bromodeoxyuridine (BrdU) IP injections (BrdU; dissolved in 0.9% saline, filtered sterile at 0.2 μm, 50 mg/kg body weight at 10 μg/ml; Sigma Aldrich, St. Louis, MO) for 7 days prior to sacrifice. See Table 1 for complete description of the design and experimental groups. At the end of the scheduled treatment, animals were sacrificed after deep anesthesia with isofluorane (Henry Schein Animal Health, OH). The dentate gyrus (DG), lateral entorhinal cortex (LEC) and gastrocnemius muscle (left and right) were collected and immediately frozen and stored at −80°C for further experiments. The subset of BrdU treated mice were deeply anesthetized with isofluorane and transcardially perfused with 0.9% saline. DG and LEC (left brain hemisphere) and gastrocnemius muscle (left and right) were collected and immediately frozen and stored at −80°C until processing for Western Blot and ELISA analysis. The right brain hemispheres were immediately stored and fixed in 4% ice cold paraformaldehyde (PFA) for 96 hours, and then equilibrated in 30% phosphate buffered sucrose for at least 48 hours. Coronal sections (40 μm) were taken sequentially through the rostral-caudal extent of the hippocampus using a freezing microtome (Thermofisher, Rockville, MD) and stored at −20°C in phosphate-buffered glycerol.

Animals were maintained in accordance with the National Institutes of Health guidelines. All protocols for procedures were approved by the NIA's Institutional Animal Care and Use Committee.

### Western blotting for AMPK pathway proteins

Frozen tissues were stored at −80°C. Samples were then thawed in ice cold RIPA Lysis buffer (Millipore Corp., Billerica, MA) completed with Protease/Phosphatase Inhibitor Cocktail (Cell Signaling Technology, Danvers, MA). Tissues were grinded using Polypropylene Pestles (Bioexpress, Kaysville, UT) and sonicated with 1 second bursts for 15 seconds at 4°C. Samples were centrifuged at 14000 g for 15 minutes at 4°C. Protein concentration was quantified via Bradford assay. Eighty micrograms of protein were boiled and separated on a 4–12% NuPAGE^®^ Bis-Tris polyacrylamide gel (Life Technologies, Frederick, MD). Proteins were then transferred to Immobilon-FL membranes (Millipore Corp., Billerica, MA), blocked with 5% BSA in TBST and treated overnight with rabbit anti-β-tubulin (1:2500, Li-Cor Biosciences, Lincoln, NE), rabbit anti-pAMPK (1:1000, Cell Signaling Technology, Danvers, MA), mouse anti-AMPK (1:500, Cell Signaling Technology, Danvers, MA), mouse anti-GLUT4 (1:1000, Cell Signaling Technology, Danvers, MA), rabbit anti-PGC-1α (1:1500, Santa Cruz Biotechnologies, Santa Cruz, CA), mouse anti-α-tubulin (1:2500, Li-Cor Biosciences, Lincoln, NE), mouse anti-IL-1β (1:800, ABCAM, San Francisco, CA), rabbit anti-VEGFa (1:1000, ABCAM, San Francisco, CA), rabbit anti-nNOS (1:1000, Cell Signaling Technology, Danvers, MA). Membranes were then tagged with 680 CW or 800 CW fluorescent goat anti-mouse or anti-rabbit IRDye (1:20, 000, Li-Cor Biosciences, Lincoln, NE), read in an OdysseyR infrared imager, and evaluated using Odyssey 2.0 software (Li-Cor Biosciences, Lincoln, NE). Precision Plus Protein Ladder (Bio-Rad, Hercules, CA) was used as a marker. Comparisons to non-perfused muscle tissue (*n* = 4 per group) were made as an additional control, and showed that saline perfusion did not affect phosphorylation and protein levels (data not shown).

### ELISA assay for BDNF quantification in dentate gyrus and lateral entorhinal cortex

BDNF quantification was performed using BDNF Emax ImmunoAssay System (Promega, Madison WI) according to manufacturer specifications. Briefly, frozen tissue were thawed in Promega lysis buffer, homogenized and then diluted 1:2.5 with ice-cold PBS; after an acidification step, total protein concentration was quantified via Bradford assay. Elisa polystyrene plates were incubated anti-BDNF monoclonal antibody (1:1000) in carbonate coating buffer before adding protein samples or standards. After being tagged with anti-human BDNF antibody (1:500) and with anti-IgY horseradish peroxidase conjugate (1:200), plates were treated with Promega TMB One Solution. To stop the reaction, 1N HCl was used. Plates were read at 450 nm with a Multiskan Ascent Plate Reader. Results were normalized against total protein amount.

### Multiplex ELISA assay for cytokines quantification in dentate gyrus and lateral entorhinal cortex

Cytokines quantification was performed using Mouse Cytokine – IR (9-plex) (Quansys Biosciences, Logan, UT) according to manufacturer specifications. Briefly, frozen tissue were thawed in ice cold PBS with Protease/Phosphatase Inhibitor Cocktail (Cell Signaling Technology, Danvers, MA), sonicated with 2 second bursts for 20 seconds and then diluted 1:2 with Quansys Sample Diluent. Samples have been loaded in duplicate on Quansys multiplex plate and incubated according to manufacturer protocol. Plate was read using OdysseyR infrared imager on channel 800. Results were analyzed using Q-view software (Quansys Biosciences). Total protein concentration was evaluated via Nanodrop (Thermo Scientific, Wilmington, DE) at 280 nm.

### BrdU immunohistochemistry and cell counts

Sections (40 μm) were stained for BrdU as previously reported [100]. Specifically, a one-in-six series of sections (240 μm apart) was pre-incubated with 0.6% H_2_O_2_ for 30 min, incubated in 2 N HCl at 37°C for 30 min, and then neutralized in 0.1 M Borate buffer at RT. After multiple washings, sections were blocked using TBS++ for 30 min at R.T., and subsequently incubated with rat anti-BrdU (1:200, Accurate Chemical, Westbury NY) overnight at 4°C. After washing in TBS and TBS++, sections were incubated for 2 h with biotin-SP-conjugated donkey anti-rat IgG (1:250, Jackson ImmunoResearch, West Grove, PA), and then washed in TBS and incubated for 2 h in ABC reagent (Vectastain Elite; Vector Laboratories, Burlingame, CA). The substrate 3, 3-diaminobenzidine (DAB; D4418, Sigma, St. Louis, MO) was applied for 5 min. BrdU-positive cells were counted through a 20 × objective (Olympus, BX51, Center Valley, PA) and multiplied by six to obtain the total number of new DG cells [101].

### Microarray analysis on dentate gyrus and lateral entorhinal cortex

Total RNA was extracted using the Trizol reagent (Invitrogen, Carlsbad, USA) according to the manufacturer's protocol. Samples RNA amount and purity were quantified using a Nanodrop (Thermo scientific, Waltham, MA); the 3 most pure and concentrated samples were chosen for microarray analysis. Samples were hybridized to MouseRef-8 v1 Expression beadchips (Illumina) following protocols listed on the Gene Expression and Genomics Unit of the NIA (http://www.grc.nia.nih.gov/branches/rrb/dna/index/protocols.htm). Micro array fluorescent signals were extracted using an Illumina BeadArray 500GX reader. The signals on each sample are normalized by log *z*-transformation to obtained *z*-scores and tests for distributions as previously described [102]. Correlation analysis, sample clustering analysis, and principal component analysis include all of the probes and were performed to identify/exclude any possible outliers. The resulting dataset was next analyzed with DIANE6.0, a spreadsheet-based microarray analysis program. After normalization by log *z*-transformation, the statistically significant gene list for each comparison condition was selected upon the conditions of (1) the probe signal detection *P*-value ≤0.02, (2) one way ANOVA over sample groups was *P*-value < 0.05, (3) pairwise *z*-test *P*-value < 0.05, (4) false discovery rates are < 0.30, and (5) gene expression changes measured by *z*-ratio were not less than 1.5 the mean of their absolute values. Further clustering/correlation analyses were done based on these selection criteria. Gene set enrichment analysis used gene expression change values (*z*-ratio) for the average of all of the genes on the microarray. Parametric analysis of gene set enrichment (PAGE) was used [103] for gene set analysis. Gene Sets include the MSIG database (http://www.broadinstitute.org/gsea/msigdb/collection_details.jsp#C2, Gene Ontology Database [http://www.geneontology.org/]; GAD human disease and mouse phenotype gene sets [103, 104] were used to explore functional level changes. The data discussed in this article have been deposited in NCBI's Gene Expression Omnibus [105] and are accessible through GEO Series accession number GEO #: GSE64607.

### RT-qPCR for illumina microarray analysis validation

Illumina microarray data were validated with RT-qPCR performed on the mRNA samples previously described. Reverse transcription was carried out on 0.5 μg of RNA using qScript cDNA Supermix (Quanta Bioscience) according to manufacturer protocol; primers for the selected genes *Tnnc1*, *Bdnf*, *Grit*, *VegfA*, and *Hsp90* were purchased from Integrated DNA Technologies using IDT online primer designer. The specific sequence of each of the primers was as follows:

*Grit*:  rev. 5′-AGAGGTATGGCATTGTGGATG-3′,
     fwd. 5′-AGGTTCTTTTGTCAGGTCGG-3′;
*VegfA*: rev. 5′-TGGTGACATGGTTAATCGGTC-3′,
     fwd. 5′-GGCAGCTTGAGTTAAACGAAC-3′;
*Tnnc1*: rev. 5′-TGTTCTTGTCACCGTCCTTC-3′,
     fwd. 5′-AGGAGCTGTCGGATCTCTTC-3′;
*Dbp*:  rev. 5′-CCATGAGACTTTTGACCCTCG-3′,
     fwd. 5′-TCATTGTTCTTGTACCTCCGG-3′;
Endogenous ctr, *Hsp90*: rev. 5′-CCTCTTTCTCACCTTT CTCTTCC-3′,
     fwd. 5′-ATTCGCAGTTCATAGGCTATCC-3′.

All primers were tested by melting curve analysis for specificity for one single amplicon product. qPCR was performed with SYBR^®^ Green PCR Master Mix (Life Technologies, Grand Island NY) on an Illumina Eco Real-Time PCR. Results were analyzed using the Comparative Ct method.

## Statistical analysis

All statistical analyses were performed using Statview (Abacus Corporation, Baltimore, MD). Analysis of immunoblotting, immunohistological and enzymatic assays results were carried out with one way analysis of variance (ANOVA). Post-hoc analysis was performed using either Fisher's test or Games-Howell's test, depending on the sample-variance and sample-size. Results are expressed as mean ± S.E.M.

## SUPPLEMENTARY FIGURE


