# Supplementary material for: Exercise-mimetic AICAR transiently benefits brain function
Source: Oncotarget. 2015 Jul 17;6(21):18293–313. doi: 10.18632/oncotarget.4715 (PMC4621892; doi:10.18632/oncotarget.4715)
Supplement: Supplementary file 1 [file oncotarget-06-18293-s001.pdf]

SUPPLEMENTARY FIGURE

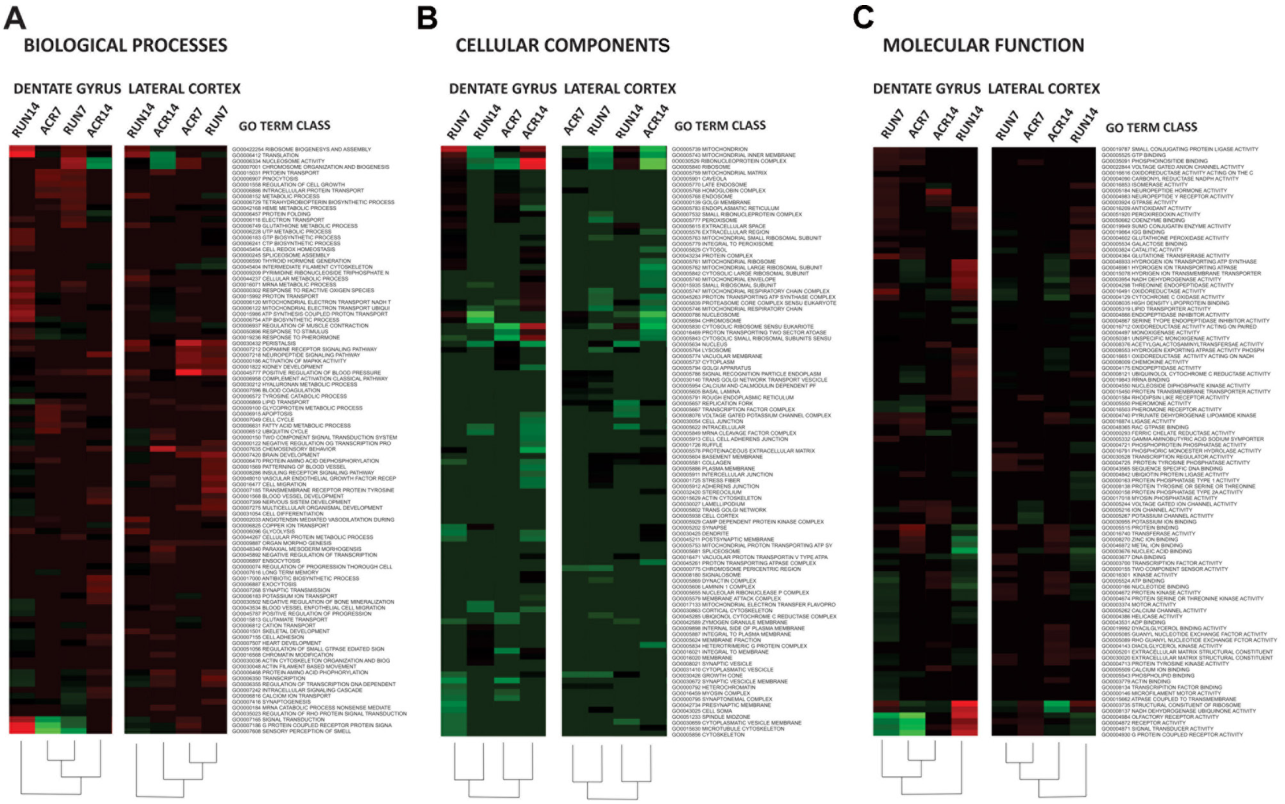

Supplementary Figure S1: Microarray analysis of Dentate Gyrus (DG) and Lateral Entorhinal Cortex (LEC):Analysis of control (CTR), AICAR treated (ACR), and voluntary running (RUN) mice after 7 and 14 days of treatment. A. Heat map of Biological Processes GO Term gene classes. **B.** Heat map of Cellular Component GO Term gene classes **C.** Heat map of Molecular Mechanisms GO Term gene classes. Up-regulated gene classes are colored in red, down-regulated gene classes are colored in green.
